# Whole-body immobilization modulates visuotactile interaction

**DOI:** 10.1007/s00221-025-07129-1

**Published:** 2025-07-12

**Authors:** Naoki Kuroda, Ryo Teraoka, Shinya Harada, Wataru Teramoto

**Affiliations:** 1 Research Organization of Open Innovation and Collaboration, Ritsumeikan University, 2-150 Iwakura-cho, Ibaraki, Osaka 567-8570 Japan; 2 Japan Society for the Promotion of Science, Kojimachi Business Center Building, 5-3-1 Kojimachi, Chiyoda-ku, Tokyo, 102-0083 Japan; 3 Faculty of Humanities and Social Sciences (Psychology), Kumamoto University, 2-40-1 Kurokami, Kumamoto, 860-8555 Japan; 4 Graduate School of Engineering, Muroran Institute of Technology, 27-1 Mizumoto-cho, Muroran, Hokkaido 050-8585 Japan

**Keywords:** Peripersonal space, Visuotactile interaction, Body immobilization, Virtual reality

## Abstract

Peripersonal space (PPS) is the space immediately around each body part. A previous study reported that hand-centered PPS shrank after hand immobilization; however, little is known about what happens in the PPS of other body parts. Here, we investigated the effect of whole-body immobilization on trunk-centered PPS by fixing the whole-body in a large box. Two experiments were conducted. Experiment 1 compared trunk-centered PPS with and without a large box. Experiment 2 manipulated the box size (large or small) to investigate the effects of box embodiment. Participants were tasked with responding as quickly as possible to a tactile stimulus on their chest while viewing a visual probe approaching from various distances. A visual facilitation effect in PPS was defined as the amount that the visual probe facilitated tactile detection in each distance condition. In addition, participants evaluated body immobilization under all conditions. The results showed that the visual facilitation effect on tactile detection was significantly greater in the with-box than in the without-box conditions in Experiment 1, and was significantly greater in the small box than in the large box conditions in Experiment 2. However, neither experiment clearly estimated PPS boundaries, although the visual probe located at farther distances had significantly less visual facilitation effect. Perceived body immobilization was stronger in the with-box than without-box conditions in Experiment 1 and was stronger in the small box than in the large box conditions in Experiment 2. These results suggest that body immobilization, rather than box embodiments, can induce stronger visuotactile interaction.

**Supplementary Information:**

The online version contains supplementary material available at 10.1007/s00221-025-07129-1.

## Introduction

The peripersonal space (PPS) is the space surrounding the body and is a body-centered margin of expanded touch representation that functions as a safety margin (Graziano and Cooke 2006). A recent study extended this to a broader concept which includes approaching (or reaching) behaviors to external objects and discussed that PPS could be related to potential action spaces to create or avoid contact between objects and the body (Bufacchi and Iannetti 2018). Rizzolatti et al. (1981a, b) first called it peripersonal space that tactile neurons representing specific body surfaces responded to a visual stimulus immediately around specific body surfaces in monkey studies. PPS representation has been reported not only in monkeys, but also in humans. PPS representation in humans was suggested in brain damage patient’s studies (e.g., di Pellegrino et al. 1997; Làdavas et al. 1998a, b) and was reported in healthy human adults (e.g., De Paepe et al. 2016; Driver and Spence 1998a, b; Salomon et al. 2017; Sambo and Forster 2009). Neurophysiological studies have shown similar brain areas in monkeys, where tactile neurons representing specific body surfaces respond to a visual stimulus nearer to the specific body surfaces (Cléry and Hamed 2018, for a review). A recent behavioral study also reported that the PPS range differed in the hand, head, and trunk (Serino et al. 2015), suggesting that the PPS can be represented in each body part.

The PPS exhibits plasticity and is modulated by body movements. Previous studies have shown that hand-centered PPS expands with hand movements (Bassolino et al. 2010) and shrinks after hand immobilization for a day (Toussaint et al. 2020). These results suggest that PPS modulation is related to body movements. Similar to the hand-centered PPS, the trunk-centered PPS expands with the body movements. Noel et al. (2015) investigated PPS modulation during treadmill walking. They used an audiotactile task (Canzoneri et al. 2012) for measuring PPS. The participants were tasked with responding to a tactile stimulus presented on their chest as soon as possible while the auditory probe approached them from the forward direction. The PPS boundary was defined as the maximum distance at which the auditory probe facilitated the tactile detection. Their results showed that the auditory probe facilitated tactile detection at a greater distance during walking than during static observation, suggesting that whole-body movement can enlarge the trunk-centered PPS. Later studies also reported trunk-centered PPS expansion with various types of self-motion information, including vision (Kuroda and Teramoto 2021), touch patterns with a walking-like sensation on the soles of the feet (Amemiya et al. 2019), and motor commands and proprioception (Kuroda and Teramoto 2022, 2025), suggesting that motor representation is strongly related to PPS expansion.

In relation to PPS modulation reflecting such body movements, the trunk-centered PPS is also known to be modulated by referring to the status of the body. Cardini et al. (2019) investigated the effects of changes over time in body size and shape on PPS modulation during pregnancy. They measured the trunk-centered PPS by presenting a tactile stimulus to the participant’s abdomen, delivered at several delays from the onset of a continuously approaching auditory probe. PPS measurements were conducted at an early stage (~ 20th week), later stage (~ 34th week), and a few weeks (~ 8 weeks) postpartum, while PPS was measured in non-pregnant women as a control group during the same period. Their results showed that while audiotactile interaction in the PPS did not significantly differ between the pregnancy and non-pregnancy groups at the early stage and the postpartum period, the range of interaction in the PPS seemed to expand more in the pregnancy group than in the non-pregnancy group at the later stage. They also reported that changes in waist size in the pregnancy group were not directly coupled with PPS modulation at a later stage. Their results suggest that the trunk-centered PPS could reflect the PPS aspect as a safety margin (Graziano and Cooke 2006) because pregnant women had an unborn baby who needed to be protected inside their bodies and additional physical load (i.e., more immobile bodies) from their fetus in the uterus. However, no previous studies have investigated the effects of whole-body immobilization itself on trunk-centered PPS modulation. Considering that the PPS can be represented in each body part (Serino et al. 2015), hand-centered PPS can be modulated by hand immobilization (Toussaint et al., 2020) and the whole body can be related to trunk-centered PPS (Noel et al. 2015); whole-body immobilization may modulate trunk-centered PPS.

The present study investigated the effect of body immobilization on the trunk-centered PPS by fixing the whole-body in a large box. Two experiments were conducted. Experiment 1 compared when the body was fixed in a large box and when the body was not fixed (without the large box). Although the results of Experiment 1 suggest that the PPS could expand more with a large box than without it, the perceived body may become larger due to the embodiment of the large box. Several previous studies have suggested that the PPS can expand up to the tip of a tool when used (Biggio et al. 2017; Maravita et al. 2002; Serino et al. 2007). To address this issue, Experiment 2 compared PPS modulation using large and small boxes.

## Experiment 1

### Methods

#### Participants

Nineteen undergraduate and graduate students (13 women; mean age: 21.2 ± 2.0 [standard deviation: SD] years) were recruited. The sample size was determined by a priori power analysis using G*power (version: 3.1.9.4). In our power analysis, we used the medium effect size as the smallest effect size of interest because we wanted to ensure replicability of the effect, if detected. The power analysis suggests that for a medium-effect size (*f* = 0.25; Cohen 1992), a sample size of 14 participants was required (α error probability = 0.05, power [1 − β error probability] = 0.80) for an analysis of variance (ANOVA) with two within-participants factors of box (2; with-box and without-box) and distance (5; nearest to farthest distances) conditions for measuring trunk-centered PPS. Finally, we adopted 19 because we expected several dropouts. Excluding five dropouts, we finally analyzed the 14 participants (12 women; mean age: 20.7 ± 1.2 [SD] years) who completed both Experiments 1 and 2. Twelve of the fourteen participants were right-handed. The Chapman handedness questionnaire was used to evaluate each participant’s dominant hand (Chapman and Chapman 1987). All participants also reported normal or corrected-to-normal vision, normal touch sensation, and no vestibular system disease. All participants were unaware of the purpose of the experiments. This study was approved by the Ethics Committee of the Graduate School of Humanities and Social Sciences, Kumamoto University, and was conducted in accordance with the principles of the Declaration of Helsinki (1964). Written informed consent was obtained from all participants before they commenced their participation.

#### Apparatus and stimuli

All the visual stimuli were presented using a head-mounted display (HMD; VIVE Pro Eye; HTC Corporation). We used our virtual reality environment, which successfully measured trunk-centered PPS in our previous study (Kuroda and Teramoto 2021). A rectangular parallelepiped tunnel consisting of left–right walls, a floor, and a ceiling (11.8 m [horizontal] × 10 m [height] × 21 m [depth]) was simulated in the virtual reality environment. The walls were covered with a square wave grating (dark grey and white pattern, each striped width: 2.1 m) perpendicular to the depth direction, which provided a depth cue. In the virtual environment, the participants viewed all the visual stimuli at 1.2 m height from the floor, 5.9 m from both sidewalls, and 1.0 m from the entrance of the tunnel. Therefore, the participants perceived themselves as being located at the center of the tunnel. A large box (color: black; size: 0.9 m^3^) was used to fix the body in a real environment, and participants were not able to move their trunks but could slightly move their legs within the box. The same size and colored boxes were simulated in the virtual reality environment. The fixation point was a red sphere (diameter: 2.0 cm) located 0.95 m above the floor and 1.8 m ahead of the participants along the midsagittal plane. To measure the trunk-centered PPS, a purple sphere (diameter: 2.0 cm) was used as the approaching probe. It was located 0.95 m above the floor. The initial position of the probe was either at 0.69 m, 1.29 m, 1.89 m, 2.49 m, or 3.09 m. The probe moved at 0.09 m/s for 1.0 s based on a previous study (Teramoto 2018), reached up to 0.6 m, 1.2 m, 1.8 m, 2.4 m, or 3.0 m, respectively, and then vanished. The probe size was presented without correction for retina size, resulting in an increased visual angle as the probe moved nearer.

To present a tactile stimulus, we utilized a vibrotactile stimulator with a 0.95 cm diameter (Vibrating Mini Motor Disc #1201; Adafruit, USA). The vibrotactile stimulator was set to vibrate at a 300-Hz frequency for 100 ms at an amplitude far above the detection threshold. As tools to measure subjective body immobilization, a white bar (size: 1.0 m [horizontal] × 0.02 m [vertical]) and a red bar as a marker (size: 0.01 m [horizontal] × 0.2 m [vertical]) were simulated in the virtual reality environment. A gamepad (Gamepad F310; Logicool) was used as the response device. All stimuli were controlled by Unity (Unity Technologies, USA; version: 2019.4.4) on a computer (GALLERIA ZG, Thirdwave Corporation) running 64-bit Windows 10.

#### Visuotactile task

We measured the trunk-centered PPS using a visuotactile procedure (Teramoto 2018) in our virtual reality system (Kuroda and Teramoto 2021). In the visuotactile procedure (Teramoto 2018), the visual probe approached the participant’s body at a constant speed (0.09 m/s) for 1 s from one of several distances and then disappeared. The participants were tasked with responding as soon as possible to tactile stimuli delivered to their chest while seeing an approaching visual probe against a static background. This paradigm was designed to minimize the effects of time expectancy (Holmes et al. 2020; Kandula et al. 2017; Noel et al. 2015) because a visual probe moved toward participants a short distance in each distance condition (no overlap in the probe trajectory between distance conditions), and each distance condition had equal tactile delays. The time expectancy effects indicate that a longer delay in tactile onset can speed up the response to the tactile stimulus. While some previous studies used the fastest RTs when only the tactile stimulus was presented as the baseline to minimize this time expectancy effect (e.g., Noel et al. 2015; Serino et al. 2015), the procedure cannot directly exclude the effect. Unlike previous studies, we independently presented a visual probe for 1 s for each distance condition. Thus, our paradigm had equal time expectancy effects across distance conditions (Kuroda and Teramoto 2021, 2022; Teramoto 2018; Teraoka et al. 2024): a nearer probe never has a higher time expectancy than a farther probe. The PPS range is defined as the distance at which the visual probe facilitates tactile detection.

### Procedure

The experimental design consisted of two within-participants variables: box condition (with-box and without-box) and probe distance condition (0.6 m, 1.2 m, 1.8 m, 2.4 m, and 3.0 m).

**Fig. 1:**
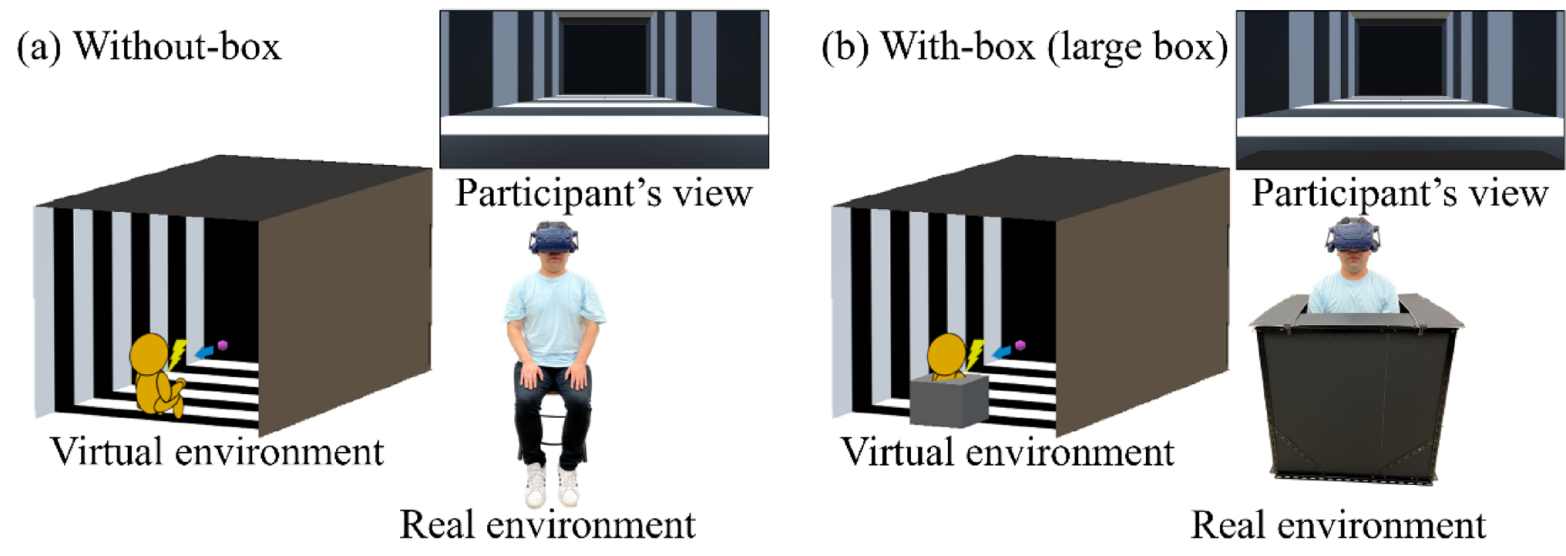
A schematic diagram of the virtual reality environment presented through a head-mounted display and the real environment. (**a**) and (**b**) indicate each of box conditions conducted in Experiment 1. In the virtual environment, a rectangular parallelepiped tunnel covered with black and white striped pattern (each striped width: 2.1 m) were illustrated. In the with-box condition, the box placed in the real environment was also visually simulated in the virtual environment. Although participants’ bodies were not presented in the virtual environment, they could see the simulated box below the field of view, allowing them to maintain the perception of being inside it. A purple sphere (probe, 0.95 m height from the floor) appeared at several distances and approached for 1 s. Participants were tasked to respond as quickly as possible to a tactile stimulus delivered to their chest with and without body immobilization by the box

The participants sat on a chair with an HMD worn on their heads. The participant’s whole-body was immobilized using the box in the with-box condition, where the chest, shoulders, and back of the participants were in contact with the box. The vibrotactile stimulator was attached to participant’s chest at 5 cm above the top of the box (the simulator height: 0.95 m above the floor; the box height: 0.9 m above the floor). In the without-box condition, participants observed visual stimuli without using a box to immobilize their bodies. In the virtual reality environment, we simulated the same size and colored box as in the real environment in each with-box condition and provided a visual cue that the observer was in a box. Participant’s body was not presented in the virtual reality environment however, participants could see the box below the field of view, allowing them to maintain the perception of being inside it (Fig. 1).

Each box condition was tested in a different session. Each session included probe and baseline trials that presented tactile stimuli with and without the probe, respectively. Baseline trials were included as a baseline tactile detection measure to clarify the visual facilitation effects and estimate the PPS boundary. At the beginning of each trial, the fixation was presented for 0.5 s, and disappeared. One second after the fixation disappearance, the probe appeared in one of distance conditions and approached the participants for 1.0 s in the probe trials, while the tactile stimulus was presented at either timing of 0.25 s, 0.5 s, or 0.75 s after the probe’s onset. In the baseline trials, the probe was not presented and the tactile stimulus was delivered with a delay of either 1.25 s, 1.5 s, or 1.75 s after the disappearance of the fixation. Thus, delays in tactile stimulation from fixation disappearance were consistent in both the probe and baseline trials. In addition to these probe and baseline trials, no vibrotactile stimulus was delivered in catch trials to avoid automatic motor responses to tactile stimuli, whereas the probe was presented in some trials and not in others. The participants were tasked with responding as quickly as possible to the tactile stimulus when presented with their dominant index finger using the response device. The next trial started after 3.0 s–4.0 s from the disappearance of the fixation point.

Each box condition had one session of 84 trials: 60 probe trials (5 distances × 3 timings × 4 repetitions), 12 baseline trials (3 timings × 4 repetitions), and 12 catch trials (with probe: 5 distances × 2 repetitions; without probe: 2 repetitions). Each trial was conducted in random order in each session. The order of the box conditions was counterbalanced across the participants.

Immediately after each session, while wearing the HMD, the participants were asked to evaluate how much they felt unable to move their body in the given experimental condition using a visual analog scale (white bar). The bar was placed 5 m ahead at the center of the display and 1 m above the floor in a VR environment. The midpoint of the bar was aligned with the midsagittal plane. The participants moved the marker horizontally using the response device. The left and right ends of the white bar indicate “easy to move” and “hard to move,” respectively. The scale was in centimeters in the virtual reality coordinates. The participant’s answer was transformed to a value (0–100: “easy to move” to “hard to move”) and was recorded. A short break was given between sessions.

Before the series of main sessions, a practice session was conducted without a box. The practice session consisted of one session of 42 trials: 30 probe trials (5 distances × 3 timings × 2 repetitions), 6 baseline trials (3 timings × 2 repetitions), and 6 catch trials (with probe: 5 distances × 1 repetition; without probe: 1 repetition). Unlike in the main session, the accuracy and reaction time (RT) (if any) of the responses were recorded immediately after each trial. All sessions took approximately 30 min.

### Statistical analysis

Analyses were performed using JASP (version 0.17.3.0; https://jasp-stats.org/), R (version 4.2.0), and MATLAB (version R2020b).

#### Analyses of visual facilitation effects

RTs in the probe and baseline trials were utilized to investigate PPS boundaries. The outlier trials were defined as those where RTs exceeded ± 2 standard deviations (SD) from the mean across all trials in each distance in each box condition. These trials were excluded from the following RT analyses and were regarded as miss trials. The tactile timings from the fixation disappearance (1.25 s, 1.5 s, and 1.75 s) were set to just prevent the participants from predicting tactile onset, so that these timings were collapsed for the analysis. Participants’ mean RTs for the probe trials (probe RTs) were calculated for each distance in each box condition. Those for the baseline trials (baseline RTs) were calculated for each box condition because they did not have distance variables. The baseline RTs were then subtracted from the probe RTs at each distance for each box condition (visual facilitation effect). In the subtracted data, positive and negative values indicate visual interference and visual facilitation effects, respectively. If the approaching probes had no effect on tactile detection, the value of the subtracted data should be zero. We defined larger visual facilitation effects as larger interactions between body and the environment in PPS (De Paepe et al. 2016; de Haan et al. 2016; Fogassi et al. 1996). In addition, the PPS boundary was defined as the farthest distance at which visual facilitation effects remained. The Shapiro–Wilk tests revealed violation of normality assumption in the visual facilitation data at several distances. To clarify the PPS boundary, we performed one-sample Wilcoxon signed-rank tests (Holm corrected) against zero for each distance in each box condition. To investigate the differences in visual facilitation effects between the box conditions, we performed a two-way repeated-measures analysis of variance (ANOVA) (box × distance) on the aligned rank-transformed (ART) data using the *ARTool* (Elkin et al. 2021; Wobbrock et al. 2011) package for R. In addition, to reveal whether the baseline RTs between box conditions were not significantly different, we conducted a Wilcoxon signed-rank test because the Shapiro–Wilk tests indicated a violation of the normality assumption in the without-box condition.

#### Analyses of perceived body immobilization

The Shapiro–Wilk tests showed normal distribution of the values for perceived immobilization in both box conditions. A paired Student *t*-test was performed.

#### Analyses of error rates and startle responses

There are two types of error trials: miss and false alarm (FA). Miss trials included trials with no response in the probe and baseline trials, and the outlier trials described above. Trials in which participants responded mistakenly in the catch trials were counted as FAs. The Shapiro–Wilk tests showed normal and violated normal distributions in the miss and FA rates, respectively. Thus, the miss and FA rates were analyzed using a paired Student *t*-test and a paired Wilcoxon signed-rank test, respectively, to investigate the differences in the speed-accuracy trade-off between the box conditions. Moreover, we analyzed d-primes calculated from miss and FA rates based on signal detection theory (Peterson et al. 1954; Tanner and Swets 1954). We performed a paired Wilcoxon signed-rank test for d-primes between the box conditions because these d-primes included FAs that violated normality assumption.

Startle response is a phenomenon in which participants are surprised at a stimulus to which their responses get faster (Graziano and Cooke 2006). We conducted the following analyses to investigate whether the differences in visual facilitation effects between the box conditions were induced by differences in startle responses. Probe presentation at the nearest distance was expected to induce the strongest startle response (Kuroda and Teramoto 2021, 2022). In general, startle responses decrease rapidly when stimuli are presented repeatedly (Graziano and Cooke 2006). Based on our previous studies (Kuroda and Teramoto 2021, 2022), the probe RTs in the nearest distance condition (0.6 m) were divided into the first and second halves of the session in each box condition and averaged for each half-session in each box condition. If startle responses strongly affected the visual facilitation effects, the first half of the RTs would be faster than the second half. The Shapiro–Wilk tests revealed violation of normality assumption in all RTs of the first and second halves of the session. We conducted a paired Wilcoxon signed-rank test between the first and second halves of the RTs for each box condition.

Holm correction was applied to multiple comparisons and denoted as adjusted *p*-values (*p*_holm_). In each analysis, effect sizes were determined using Cohen’s *d* (absolute value), Cliff’s *d* (absolute value), or partial Eta squared (η_p_^2^).

### Results and discussions

**Fig. 2:**
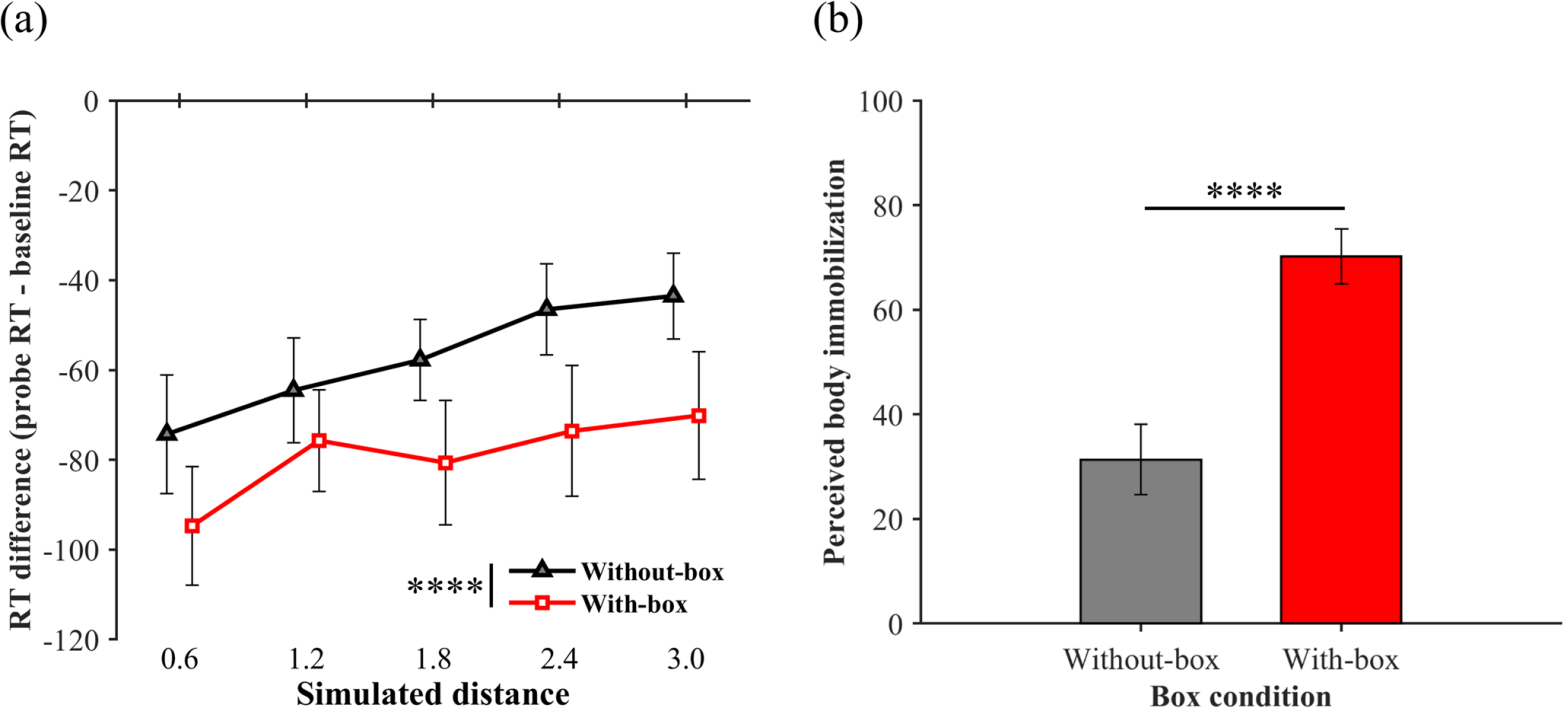
Results of the visual facilitation effects (**a**) and perceived whole-body immobilization (b**)** in Experiment 1. **a** Each plot represents an average of visual facilitation effects across participants as a function of distance in each box condition. Each distance function was illustrated slightly lateral shifted for a visibility purpose. The values were calculated by subtracting the baseline RTs from the probe RTs in each distance in each box condition. Negative and positive values indicate visual facilitation and interference effects, respectively. **b** Each bar graph represents an average of perceived whole-body immobilization across participants in each box condition. Larger values indicate that participants perceive to be more immobile. Error bars indicate standard errors. ****p < .001

Raw data (anonymized) are available at Open Science Framework (https://osf.io/5dvek/). Figure 2a shows the visual facilitation effect as a function of the distance for each box condition (see a supplementary file for individual plots). One-sample Wilcoxon signed-rank tests revealed significant visual facilitation effects for all distance conditions in the with-box [*V*s < 1.01, *p*_*holm*_ < 0.001] and without-box conditions [*V*s < 0.01, *p*_*holm*_ < 0.001]. These results suggest that the PPS boundary in both conditions is outside the range measured in this study. A two-way ANOVA with ART revealed significant main effects of box [*F* (1, 117) = 31.86, *p* < 0.001, η_p_^2^ = 0.21] and distance [*F* (4, 117) = 7.10, *p* < 0.001, η_p_^2^ = 0.20], but no interaction [*F* (4, 117) = 0.35, *p* = 0.841, η_p_^2^ = 0.01]. The visual facilitation effect was significantly greater in the with-box condition than in the without-box condition. A multiple comparison for the main effect of distance revealed a significant difference between the 0.6 m and other distance conditions, suggesting that the visual facilitation effect decreased with increasing distance. As for baseline RTs, a Wilcoxon signed-rank test revealed non-significant differences between the box conditions [*V* = 26, *p* = 0.100, Cliff’s *d* = 0.13], suggesting that the box had no effect on the tactile-only responses.

Figure 2b shows the mean perceived body immobilization across participants for each box condition. A paired Student *t*-test revealed significantly perceived immobility in the with-box than without-box conditions (*t* (13) = 6.15, *p* < 0.001, *d* = 1.64) so that our manipulation using the box succeeded.

The miss rates (± SD) were 6.2% (± 0.6%) and 5.2% (± 0.6%) in the with-box and without-box conditions, respectively. The FA rates were 0.6% (± 0.6%) and 1.8% (± 1.3%) in the with-box and without-box conditions, respectively. A paired Student *t*-test and paired Wilcoxon signed-rank test revealed no significant differences between the box conditions for miss rates [*t* (13) = 1.11, *p* =.286, *d* = 0.43] and FA rates [*W* = 4.50, *p* =.586], respectively. For d-primes, a paired Wilcoxon signed-rank test revealed no significant differences [*W* = 47.50, *p* =.500]. Thus, the effect of the speed-accuracy trade-off on the difference between the box conditions was negligible. In addition, paired Wilcoxon signed-rank tests revealed no significant differences between the RTs in the first and second halves of the session in each box condition [*W*s > 27.99, *p*s >.134], suggesting that the differences in startle responses between the box conditions cannot fully explain the difference in the visual facilitation effect between them.

The results of Experiment 1 suggest that the visuotactile interaction can be stronger when it is difficult to move the whole-body. The main effect of the box was significant, with visual facilitation effects being greater in the with-box than without-box conditions. Furthermore, the main effect of distance was also significant, as visual facilitation effects decreased at farther distances. In addition, there was no significant difference in baseline RTs between the box conditions. Although we did not clearly estimate PPS boundary, taken together the significant main effects and the non-significant baseline RTs, support the speculation that PPS boundary might be located at a farther distance in the with-box than without-box condition. The participants perceived themselves as more immobile when restricting their whole-body movements using the box, suggesting that body immobilization affected visuotactile facilitation. However, another interpretation would be also raised. There is a possibility that the embodiment of the box could have been induced, and the visuotactile interaction might have been stronger, corresponding to the box itself. Previous studies have reported that when humans continue to use a tool (e.g., a stick) with their hand, they take the tool into their bodies (Maravita et al. 2002). This phenomenon can be induced in virtual environments (D’Angelo et al. 2018). Although these findings assume active tool use, studies on rubber/mirror hands in the real environment suggest that a tool could be embodied not by actively using it but just by seeing it (Holmes and Spence 2005; Litwin et al. 2020).

## Experiment 2

Experiment 1 revealed that the visuotactile facilitation effects were significantly larger during whole-body immobilization. However, it is not clear whether the visuotactile interaction increased by body immobilization or the embodiment of the box. Although it is well known that tool embodiment is little induced when a tool is not similar to body parts (Bertamini and O’Sullivan 2014; Finotti et al. 2023), to further investigate box embodiment in the present study, Experiment 2 addressed this issue using small and large boxes for body immobilization. If the visuotactile interaction was modulated by the box embodiment, visual facilitation effects should be larger with a large box than with a small box.

### Methods

#### Participants

Fourteen participants (12 women; mean age: 20.7 ± 1.2 [SD] years) who took part in Experiment 1 completed all tasks in Experiment 2.

##### Apparatus and stimuli

The same apparatus and stimuli were used as in Experiment 1, except for the following. In addition to the large box (color: black; size: 0.9 m^3^) used in Experiment 1, to immobilize the body more tightly, a small box (color: black; size: 0.75 m [horizontal] × 0.9 m [vertical] × 0.75 m [depth]) was utilized in the real environment, and participants were not able to move their legs in addition to their trunks. Boxes of the same size and color were simulated in a virtual reality environment.

#### Procedure

**Fig. 3:**
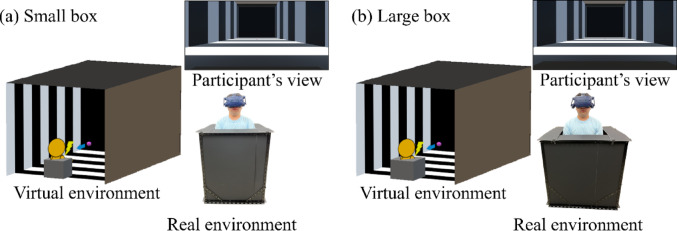
A schematic diagram of the virtual reality environment presented through a head-mounted display and the real environment. (**a**) and (**b**) indicate each of box conditions conducted in Experiment 2. In the virtual environment, a rectangular parallelepiped tunnel covered with black and white striped pattern (each striped width: 2.1 m) were illustrated. In each box condition, the box placed in the real environment was also visually simulated in the virtual environment. Although participants’ bodies were not presented in the virtual environment, they could see the simulated box below the field of view, allowing them to maintain the perception of being inside it in each box condition. A purple sphere (probe, 0.95 m height from the floor) appeared at several distances and approached for 1 s. In the real environment, participants were tasked to respond as quickly as possible to a tactile stimulus delivered to their chest with and without body immobilization by the box

The same procedure as Experiment 1 was followed, except for the following: The without-box condition was replaced by the small box condition; thus, the large and small box conditions were compared as independent variables in Experiment 2 (Fig. 3). Participants completed Experiment 2 within a maximum of 45 days following Experiment 1 (mean days: 11.8 ± 1.2 [SD]), depending on their availability.

### Statistical analysis

The same statistical analyses as Experiment 1 were conducted. In addition, to further investigate the visual facilitation effects during body immobilization across the experiments, we conducted an exploratorily analysis for the baseline RTs, which were compared using one-way ANOVA (four box conditions across the experiments) with ART.

### Results and discussions

**Fig. 4:**
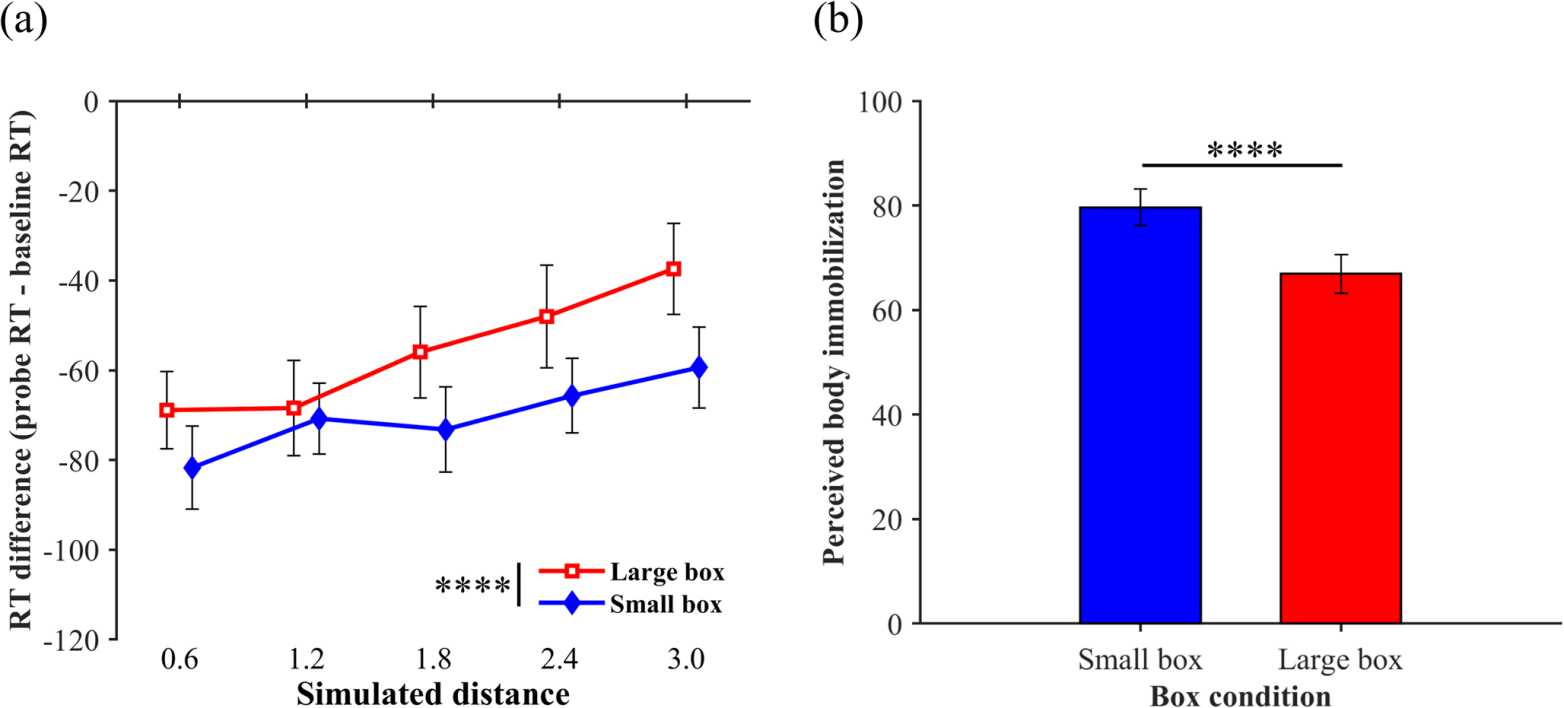
Results of the visual facilitation effects (a) and perceived whole-body immobilization (b) in Experiment 2. **a** Each plot represents an average of visual facilitation effects across participants as a function of distance in each box condition. Each distance function was illustrated slightly lateral shifted for a visibility purpose. The values were calculated by subtracting the baseline RTs from the probe RTs in each distance in each box condition. Negative and positive values indicate visual facilitation and interference effects, respectively. **b** Each bar graph represents an average of perceived whole-body immobilization across participants in each box condition. Larger values indicate that participants perceive to be more immobile. Error bars indicate standard errors. ****p < .001

Raw data (anonymized) are available at Open Science Framework (https://osf.io/5dvek/). Figure 4a shows the visual facilitation effect as a function of the distance for each box condition (see a supplementary file for individual plots). One-sample Wilcoxon signed-rank tests revealed significant visual facilitation effects for all distance conditions in the large box [*V*s < 8.01, *p*_*holm*_ <.004] and small box conditions [*V*s < 0.01, *p*_*holm*_ <.001]. These results suggest that the PPS boundary in both conditions is outside the range measured in this study. A two-way ANOVA with ART revealed significant main effects of box [*F* (1, 117) = 14.07, *p* <.001, η_p_^2^ = 0.11] and distance [*F* (4, 117) = 4.86, *p* =.001, η_p_^2^ = 0.14], but no interaction [*F* (4, 117) = 0.59, *p* =.673, η_p_^2^ = 0.02]. The visual facilitation effect was significantly greater in the small box condition than in the large box condition. A multiple comparison for the main effect of distance revealed a significant difference between 0.6 m and 2.4 m, between 0.6 m and 3.0 m, and between 1.2 m and 3.0 m, suggesting that the visual-facilitation effect decreased as a function of distance. As for baseline RTs, a Wilcoxon signed-rank test revealed non-significant differences between the box conditions [*V* = 70, *p* =.094, Cliff’s *d* = 0.15], suggesting that the box had no effect on the tactile-only responses.

Figure 4b shows the mean perceived body immobilization across participants for each box condition. A paired Student *t*-test revealed a significant perception of immobility in the small box condition than in the large box condition (*t* (13) = 5.52, *p* <.001, *d* = 1.48), indicating that our manipulation using the boxes succeeded.

The miss rates (± SD) were 5.7% (± 0.5%) and 5.6% (± 0.8%) in the large and small box conditions, respectively. The FA rates were 1.8% (± 1.0%) and 0.6% (± 0.6%) in the large and small box conditions, respectively. A paired Student* t*-test and paired Wilcoxon signed-rank test revealed no significant differences between the box conditions for miss rates [*t* (13) = 0.15, *p* =.883, *d* = 0.30] and FA rates [*W* = 2.50, *p* =.424], respectively. For d-primes, a paired Wilcoxon signed-rank test revealed no significant differences [*W* = 63.00, *p* =.234]. Thus, the effect of the speed-accuracy trade-off on the difference between the box conditions was negligible. In addition, paired Wilcoxon signed-rank tests revealed no significant differences between the RTs in the first and second halves of the session in each box condition [*W*s > 30.99, *p*s >.193], suggesting that the differences in startle responses between the box conditions cannot fully explain the differences in the visual facilitation effects between them.

As an exploratorily analysis, we compared the baseline RTs between the experiments. A one-way ANOVA with ART revealed a non-significant main effect [*F* (3, 39) = 2.09, *p* =.117, η_p_^2^ = 0.14], suggesting that the box had no effect on the tactile-only response across the experiments.

The results of Experiment 2 suggest that the visuotactile interaction can be stronger in the small box condition, where participants perceived themselves as more immobile, compared to the large box condition. If embodiment of a larger box could enhance visuotactile interaction, then the interaction should be greater in the large box than in the small box condition. The results of Experiment 2 contradicted this hypothesis, suggesting that not box embodiment but the perception of whole-body immobilization contributes to the modulation of the visuotactile interaction. In addition, the exploratory analysis demonstrated that baseline RTs did not differ significantly between any pairs of box conditions, suggesting that the visual facilitation effect itself was modulated in each box condition across the experiments.

## General discussion

The present study investigated the effects of whole-body immobilization on trunk-centered PPS representation using boxes that restricted participants’ movements. Although both experiments did not clearly estimate PPS, however, Experiment 1 showed that the visual facilitation effect and the rating for body immobilization were larger in the with-box condition than in the without-box condition. Experiment 2 similarly showed that the effect and rating were greater in the small box condition than in the large box condition. These results suggest that perceived whole-body immobilization can modulate the visuotactile interaction.

It is well known that our perception is modulated by various sensory inputs, including individual factors such as physical load, age, degree of fatigue, and health (Proffitt 2006). Bhalla and Proffitt (1999) showed that individual factors (i.e., physical fitness and age) made hills perceive as steeper. They also reported that the hills appeared steeper when people were fatigued or wearing a heavy backpack. The latter one suggests that whole-body immobilization can affect visual perception. In another study, Vagnoni et al. (2017) showed that time-to-collision was perceived as shorter when whole-body movements were temporarily restricted by a chin rest. Thus, body immobilization is an important factor that modulates the interactions between humans and the external environment.

The PPS is represented differently in each body part (e.g., hand, head, and trunk) (Serino et al. 2015). In a hand-centered PPS study, Toussaint et al. (2020) reported that short-term upper-limb immobilization for one day shrank the reachable space, suggesting that a decrease of potential actions by the hand can cause changes in hand PPS representation. Lourenco and Longo (2009) also measured the hand-centered PPS when hand movements were restricted by wrist weight. They reported that the PPS shrank with the wrist weight compared to that without it. This suggests that the online status of the hands can contribute to the modulation of the hand-centered PPS. In contrast, Vagnoni et al. (2017) reported that head immobilization using a chin rest did not affect hand-centered PPS. This suggests that the hand-centered PPS does not change when the action restrictions are irrelevant to the body part. These findings support Bufacchi and Iannetti’s (2018) action field theory of the PPS, which states that PPS representation can change depending on the potential actions.

The novelty of the present study is that the visuotactile facilitation was modulated by referring to the online status of whole-body immobilization with the box. Previous studies have shown that the trunk-centered PPS is strongly associated with whole-body movements (Kuroda and Teramoto 2022; Noel et al. 2015). Cardini et al. (2019) investigated trunk-centered PPS in pregnant women who experienced changes in body size and shape over several months and reported that the PPS seemed to expand more in the pregnancy group than in the non-pregnancy group. This PPS expansion can be explained not only by decreased mobility due to an additional load (unborn baby) but also by changes in motivation, such as protecting their unborn baby from external dangers. However, it was not clear whether body immobilization itself affected PPS expansion. The results demonstrated that greater tactile facilitation on the chest by the approaching visual probe was observed when the whole-body was perceived as more restricted. In general, visuotactile interaction is stronger in PPS, and become significantly stronger when an approaching probe is located closer to the body (De Paepe et al. 2016; de Haan et al. 2016). If the results that visuotactile facilitation was stronger when perceiving body immobilization more strongly could represent PPS expansion, the direction of modulation in PPS found in this study could be inconsistent with that found in studies investigating the modulation of hand-centered PPS by decreased hand movability (Lourenco and Longo 2009; Toussaint et al. 2020): expansion in the former and shrinkage in the latter studies. The non-significant baseline results supported the speculation of PPS expansion during body immobilization. We believe that our results might be explained by the action field theory of PPS (Bufacchi and Ianneti 2018). Specifically, this theory holds that the range of PPS reflects the behavioral relevance of not only actions aimed at creating contact between objects and the body, but also those aimed at avoiding contact between them. We asked participants to respond quickly to tactile stimuli while presenting approaching stimuli. This situation might cause the brain to weigh avoidance actions more than reaching actions.

The present study has several limitations. First, characteristics of the stimuli in our virtual reality environment had a less realistic background and a less threatening probe. The lack of a realistic background could have affected depth perception, resulting in unclear PPS estimation. In addition, if an approaching object is perceived as a threat (e.g., a bee or a knife), its relationship to the defensive margin should be defined more clearly. Second, physical and visual body immobilization were not divided. The visual boxes continued to be presented during the session in the virtual environment to make the participants aware of the immobilization of their whole body. However, it is unclear how the visual box affected the results. Finally, the effect of the participant’s sex is raised. Many female participants (12 out of 14) took part in the study. In general, females are more likely to engage in avoidance behaviors than males in daily life. If this speculation is correct, then the visuotactile interaction in males may be smaller than that in female. A previous study suggested that females maintained greater distances from objects and human avatars than males in the virtual environment (Iachini et al. 2014). Thus, future studies should address the effects of sex on body immobilization.

## Conclusion

This study investigated the effects of whole-body immobilization on trunk-centered PPS representations. The results did not clearly estimate PPS boundary but showed that visual facilitation effects were greater in the with-box than without-box conditions in Experiment 1 and were greater in the small box than in the large box conditions in Experiment 2. These results are consistent with the perception of whole-body immobilization reported by the participants. These results suggest that whole-body immobilization could induce stronger visuotactile interaction.

## Electronic supplementary material

Below is the link to the electronic supplementary material.

Supplementary Material 1

## Data Availability

The datasets generated and analyzed in the present study are available at https://osf.io/5dvek/.
