# Supplementary figures and images for: Whole-body immobilization modulates visuotactile interaction
Source: Exp Brain Res. 2025 Jul 12;243(8):186. doi: 10.1007/s00221-025-07129-1 (PMC12255558; doi:10.1007/s00221-025-07129-1)

Individual data in Experiment 1

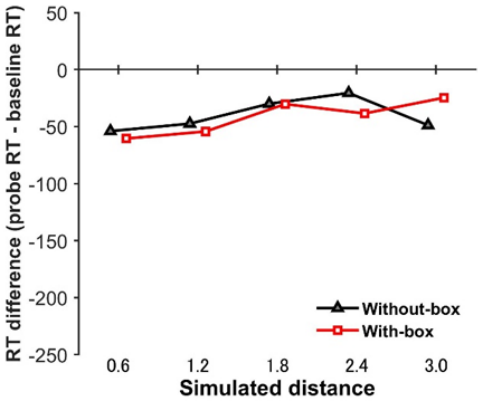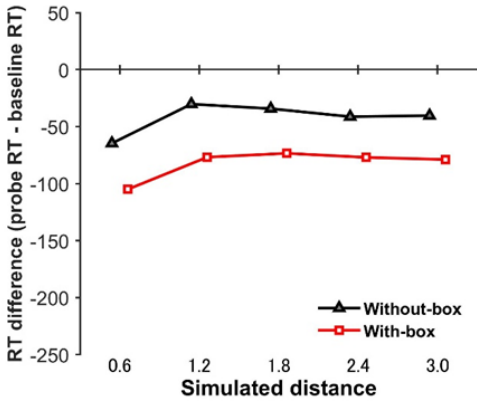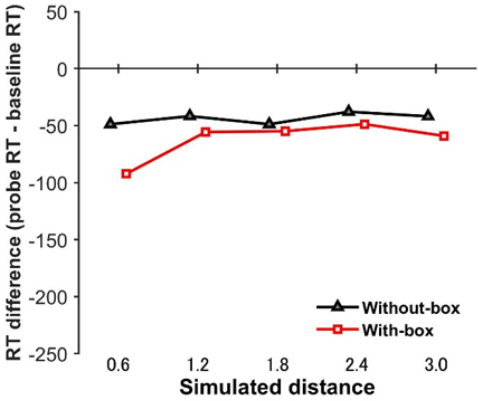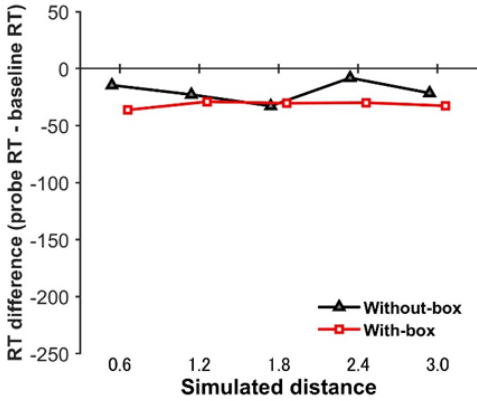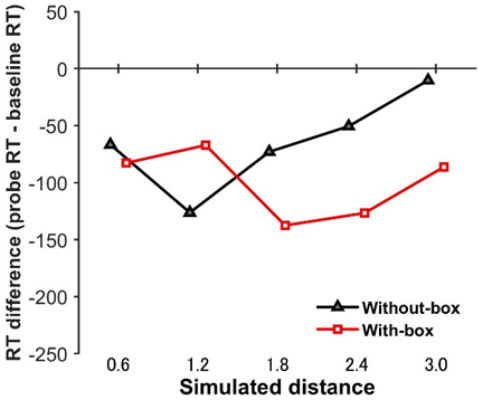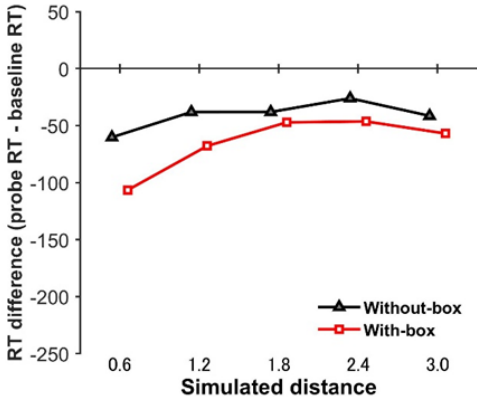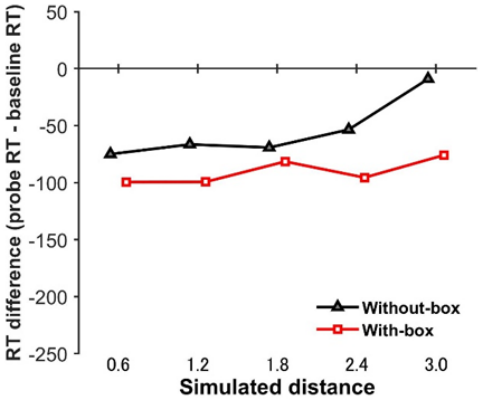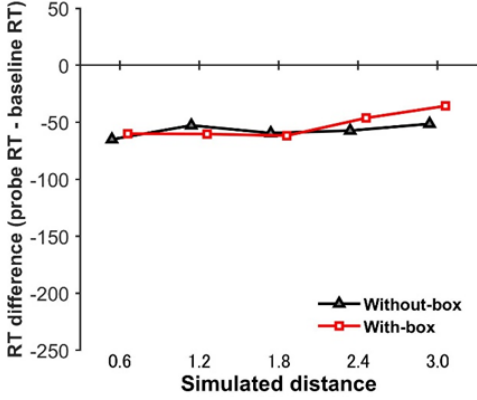

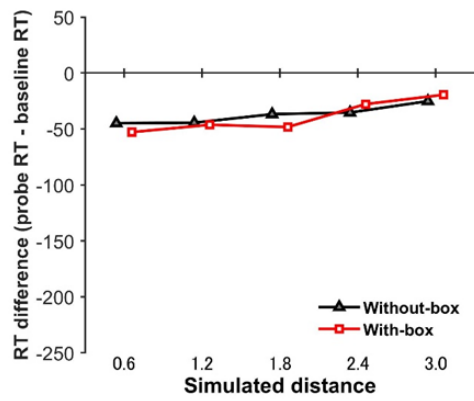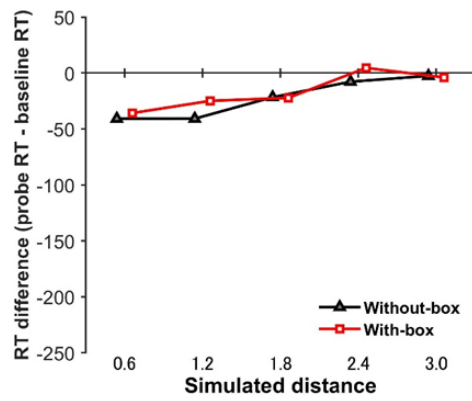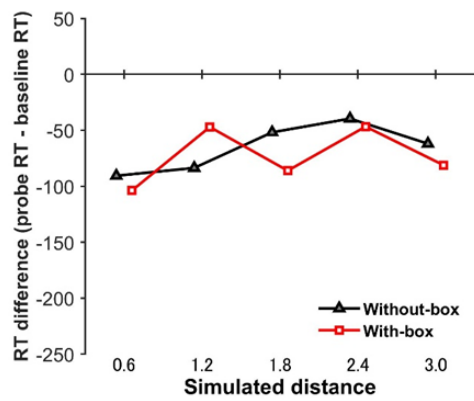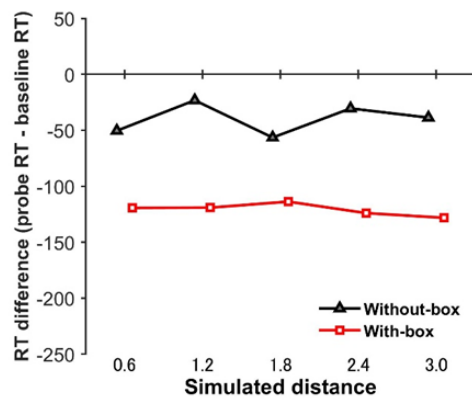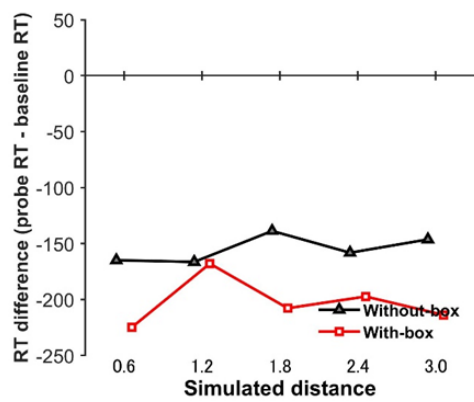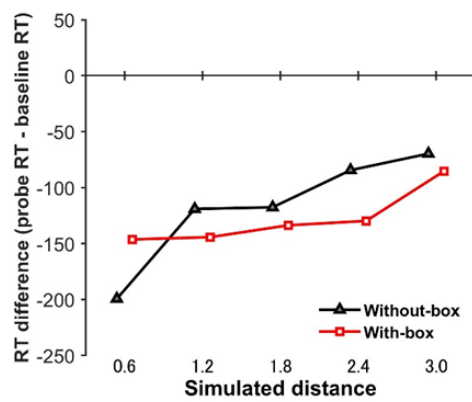

Individual data in Experiment 2

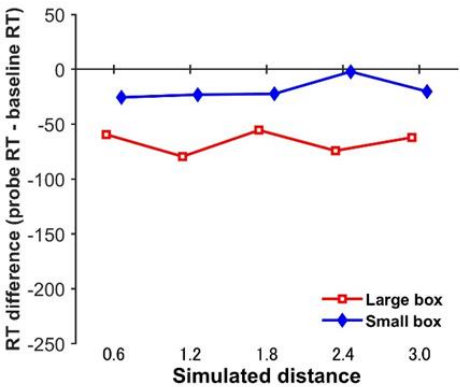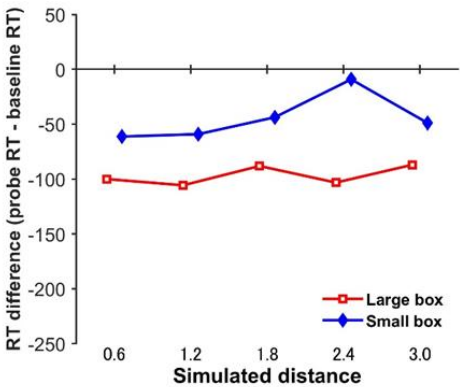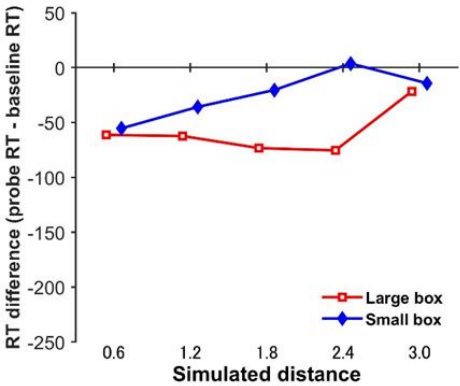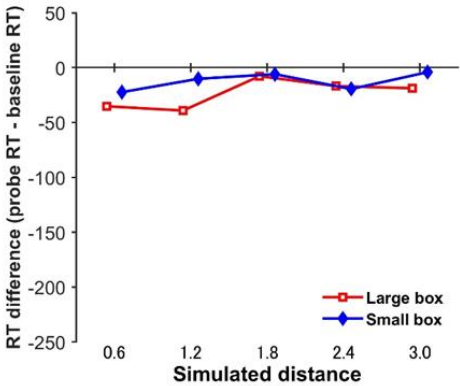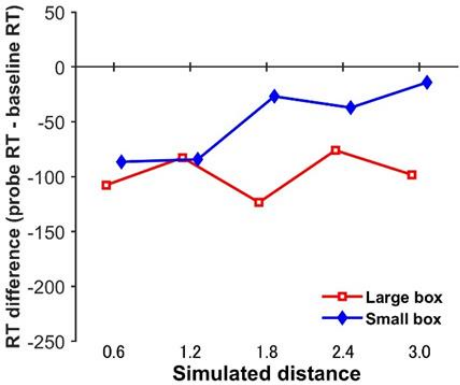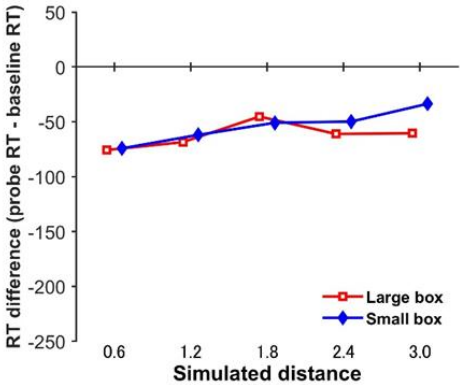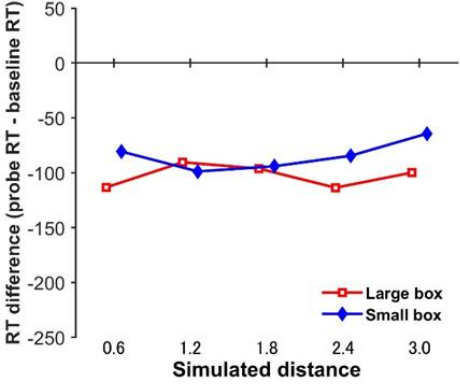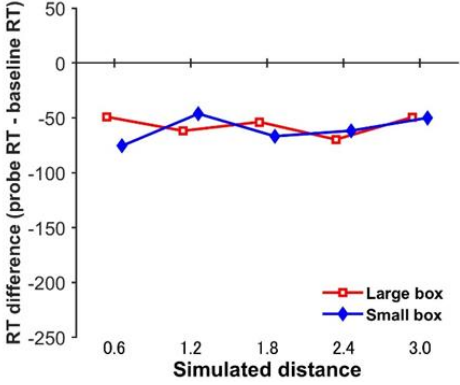

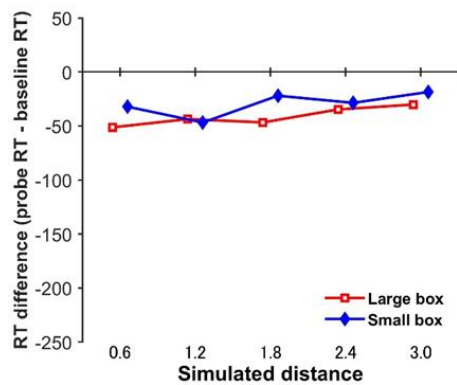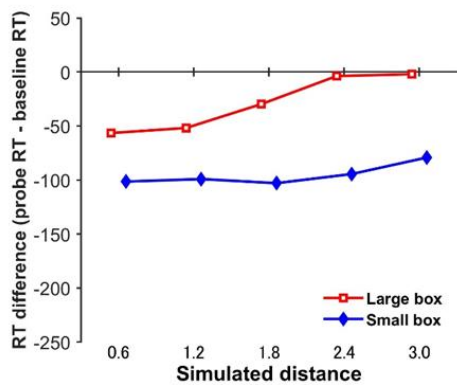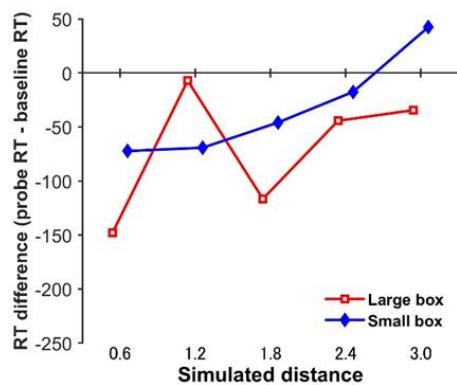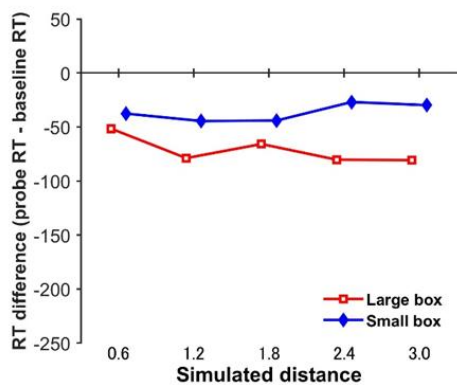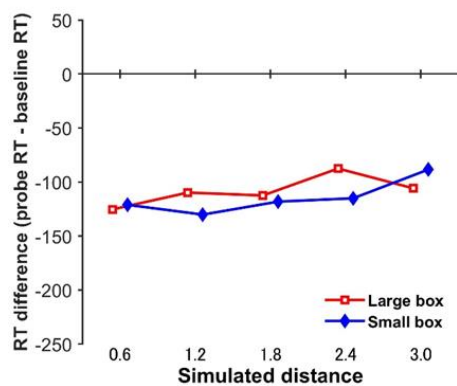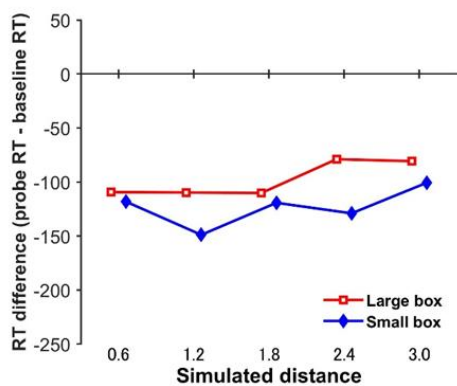

Supplement: Supplementary file 1 — Supplementary Material 1 [file 221_2025_7129_MOESM1_ESM.pdf]
